# Factor structure of the Social Phobia Scale (SPS) and the Social Interaction Anxiety Scale (SIAS) in a clinical sample recruited from the community

**DOI:** 10.1186/s12888-023-05142-8

**Published:** 2023-09-04

**Authors:** Dajana Šipka, Jeannette Brodbeck, Ava Schulz, Timo Stolz, Thomas Berger

**Affiliations:** 1 Department of Clinical Psychology and Psychotherapy, University of Bern, Fabrikstrasse 8, 3012 Bern, Switzerland; 2 School of Social Work, University of Applied Sciences and Arts Northwestern Switzerland, Olten, Switzerland

**Keywords:** Social anxiety, Social phobia scale, Social interaction anxiety scale, Factor analysis, Bifactor model

## Abstract

**Background:**

The Social Phobia Scale (SPS) and the Social Interaction Anxiety Scale (SIAS) are widely used self-report questionnaires to assess symptoms of social anxiety. While SPS measures social performance anxiety, SIAS measures social interaction anxiety. They are mostly reported simultaneously, but there have not been consistent results of the joint factor structure and therefore no consistent recommendations on how to use and evaluate the questionnaires. This study aimed (1) to evaluate the underlying joint factor structure of the SPS and SIAS and (2) to test whether SPS and SIAS are reliable scales to assess two different aspects of social anxiety.

**Methods:**

The one-factor, two-factor, and bifactor models were tested in a clinical sample recruited from the community and diagnosed with a social anxiety disorder. Exploratory and confirmatory factor analyses were conducted, bifactor-specific indices were calculated, and the content of the less fitting items was examined.

**Results:**

Confirmatory factor analyses showed that the best-fitting model was the bifactor model with a reduced set of items. The bifactor-specific indices showed that the factor structure cannot be considered unidimensional and that SPS and SIAS are reliable subscales. A closer examination of the less fitting item content and implications for future studies are discussed.

**Conclusions:**

In conclusion, SPS and SIAS can be reported together as an overall score of social anxiety and are separately reliable measures to assess different aspects of social anxiety.

**Trial registration:**

This is a secondary analysis of data from two trials registered under ISRCTN75894275 and ISRCTN10627379.

**Supplementary Information:**

The online version contains supplementary material available at 10.1186/s12888-023-05142-8.

## Background

Social anxiety is a common anxiety that many people experience in their day-to-day social life. The range of feared situations can be broad, from difficulty making eye contact to giving a presentation in front of others. Over the years, there have been many categorizations of social anxiety (e.g., [1]). Still, the core fear of all these categories is the fear of negative evaluation by others and not being able to maintain a favorable impression of oneself [2]. The Social Phobia Scale (SPS) and the Social Interaction Anxiety Scale (SIAS) are widely used self-report questionnaires to assess social anxiety symptoms and are often reported together [3]. They both claim to measure different aspects of social anxiety: While SPS measures social performance anxiety, SIAS measures social interaction anxiety [4].

Even though the questionnaires are widely used in research and in clinical practice, there are no consistent recommendations on how to use and assess the questionnaires. There have been two different recommendations: Gomez & Watson [5] and Thompson et al. [3] recommended, based on non-clinical samples, to use the SPS and SIAS only simultaneously for the assessment of general social anxiety (i.e., report the total sum of both questionnaires), but not separately for the assessment of social performance anxiety by SPS respectively the social interaction anxiety by SIAS (i.e., report the separate sum score of each questionnaire). By contrast, based on clinical samples, Heimberg et al. [4] and Heidenreich et al. [6] recommended using the SPS and SIAS independently of each other to assess and report social performance and social interaction anxiety separately. One possible explanation for the inconsistent recommendations may be that the two aspects of social anxiety only exist distinctly at a clinical level [3].

These recommendations were all made based on the results of factor analyses. Exploratory factor analysis (EFA) and confirmatory factor analysis (CFA) are possible methods to determine the joint factor structure and draw recommendations on how to use the two questionnaires in practice. Evidence regarding the joint factor structure of the SPS and the SIAS is inconsistent. Gomez & Watson [5] found a bifactor solution with SPS and SIAS as group factors, while almost all items loaded on an additional general factor. By contrast, Safren et al. [7] found a three-factor solution, with one general factor being a higher-order factor. Carleton et al. [8] found a three-factor solution as well, without a higher-order factor but with one SIAS factor and two SPS factors. Heidenreich et al. [6] on the other hand found a two-factor solution, SPS and SIAS, but suggested due to the high correlation (*r* = 0.69) “[…] that both scales represent facets of a higher-order construct.” (p.583). Most studies that look at the questionnaires’ factor structure, use uni- or multi-dimensional models and do not test for the bifactor model (cf. previous studies listed by Eidecker et al. [9] or Gomez & Watson [5]). This study provides a more complex and relatively new factor structure analysis than most previous studies since the current study simultaneously compared the one-factor, two-factor, and bifactor models in a clinical sample from the community. Additionally, with the bifactor-specific indices, it is possible to analyze further the resulting subscales and their reliability (see 2.5. Factor Analyses: Models).

Multiple aspects make the comparison between different factor analyses studies using these two questionnaires challenging: Some studies compare the structure of SIAS with the structure of SPS separately (e.g., [10]) or only assess one of the questionnaires (mostly SIAS, e.g., [9, 11, 12]), even though in practice the two questionnaires are often used together. Most studies do not use clinical samples (i.e., participants with a social anxiety disorder (SAD) diagnosis), but the general population or student samples. Rodebaugh et al. [11] compared the SIAS structure of clinical and undergraduate student samples. They found slight but systematic differences in the responses and suggested a separate cut-off score for the student sample. Carleton et al. [8] tested the joint factor structure of SPS and SIAS with a student and a clinical sample and found the same three-factor solution for both samples but included only 14 out of the overall 40 items in the final model. As Kupper & Denollet [10] summarized, the items may have different meanings for the clinical and the non-clinical samples.

Depending on the resulting factor model, different implications can be drawn based on the model: A one-factor model would support using an overall sum score of both questionnaires to assess general social anxiety, but it would not support using both questionnaires to assess distinct aspects of social anxiety (i.e., social performance and interaction anxiety) through two separate sum scores of each questionnaire. A two-factor model would support the distinction of the two social anxiety aspects by calculating each sum score separately, but not the joint use of the questionnaires to assess general social anxiety by calculating the overall sum score. A validly proven bifactor model with good bifactor-specific indices would firstly, support evaluating general social anxiety by calculating the overall sum score and would secondly, support using both questionnaires separately to assess social performance and interaction anxiety as distinct aspects of social anxiety by calculating each sum score separately. As Thompson et al. [3] pointed out, the question about the different aspects of social anxiety is especially interesting since the 5th edition of the Diagnostic and Statistical Manual of Mental Disorders (DSM-5; [13]) has introduced the performance anxiety category within the social anxiety disorder diagnosis.

## Methods

### Aims

The two aims of this study were 1) to assess the joint factor structure of SPS and SIAS in a clinical sample since there are only a few studies using clinical samples and 2) to test whether SPS and SIAS are reliable scales to assess two different aspects of social anxiety beyond the assessment of social anxiety in general. Two clinical samples from two studies were used. After testing whether the two samples could be combined, the joint factor structure of SPS and SIAS was tested in a combined sample using CFA, EFA, bifactor model-specific indices and a content examination of the less fitting items in the final model. Studies usually do not thoroughly examine the less-fitting item content but rather report only the statistical characteristics (e.g., item loadings). By examining the content, one might find a common theme and might indicate not only that the items are not fitting, but also why they are not. The following models were tested: one-factor, two-factor, and bifactor model.

### Measures

#### Social Phobia Scale and Social Interaction Anxiety Scale

The Social Phobia Scale (SPS; [14]; German Version: [15]) is a self-report questionnaire that assesses social anxiety in performance-related, routine situations and activities (e.g., eating, writing in front of others, etc.). The Social Interaction Anxiety Scale (SIAS; [14]; German Version: [15]) is a self-report questionnaire that assesses social anxiety in interactional situations (e.g., conversations with strangers, friends, etc.). Both questionnaires contain 20 items each. Response categories are from 0 = “not at all” to 4 = “extremely”, with a total sum score ranging from 0 to 80 per questionnaire, respectively, from 0 to 160 for both questionnaires combined. Items 5, 9, and 11 in the SIAS are reversed scored. The internal consistency of the German versions (which were used in the samples of this study) for the SAD patient population is excellent, with $\alpha =0.94$ for each questionnaire [15]. The internal consistency for the current combined sample in this study is lower but still excellent, with $\alpha =0.91$. See Supplementary file 1 for the item wording.

### Participants

Two samples from two different studies were used, namely from Schulz et al. ([16], sample (a)) and Stolz et al. ([17], sample (b)). Both studies recruited participants from the general population through newspaper articles, interviews on radio and TV, and online forums in Switzerland, Germany, and Austria. Participants could download the study information (where participants were informed about the whole study procedure) and the informed consent form (ICF) from the respective website and send back the signed ICF via e-mail or post. Both studies established a SAD diagnosis by using the Structured Clinical Interview for DSM-IV – Axis I disorder (SCID; [18]). Schulz et al. [16] conducted a randomized controlled trial (RCT) comparing the efficacy of an internet-based cognitive behavioral therapy (ICBT) program for SAD between a clinician-guided group ICBT with clinician-guided individual ICBT, and a waitlist control group (WL). The final sample included 149 participants with a SAD diagnosis. Stolz et al. [17] used a three-armed RCT for comparing the efficacy of an ICBT program for SAD between three groups using the program as a mobile version, as a computer version, and a WL. The final sample included 150 people with a SAD diagnosis.

The inclusion criteria for both studies were a cut-off score on the SPS of > 22 or on the SIAS of > 33, a SAD diagnosis according to the SCID, an age of at least 18 years, access to a computer with internet connection, sufficient mastery of the German language, and no psychotherapy during study participation. Exclusion criteria were active suicidal plans, a history of bipolar or psychotic disorders, and no change in psychiatric medication during the previous month (if psychiatric medication intake was present). Both studies worked with the same ICBT program for SAD [19, 20]. For this study, only the baseline data was used. All items were answered without any missing data apart from one participant who was excluded as no detailed item data were available.

### Sample comparison

To test whether both samples could be combined, the samples’ socio-demographic data (i.e., age, sex, education, and relationship status), as well as item responses, were compared. The program R Studio (Version 2023.6.1.524) was used to compare the two samples. Shapiro tests showed no normal distribution for age nor for any of the item responses per sample (SPS 1–SPS 20, SIAS 1–SIAS 20) (*p* < 0.001). This is because age showed a clear right-skewed distribution as 62% of the sample was between 18 to 35 years of age within a total range of 18 to 76 years. Therefore, non-parametric tests were used for all comparisons. The age difference was calculated with the Mann–Whitney U test. Since the other socio-demographic data were dummy coded, Chi-square tests were used. For the item response comparisons, multiple Mann–Whitney U tests were used.

### Factor analyses: models

**Fig. 1:**
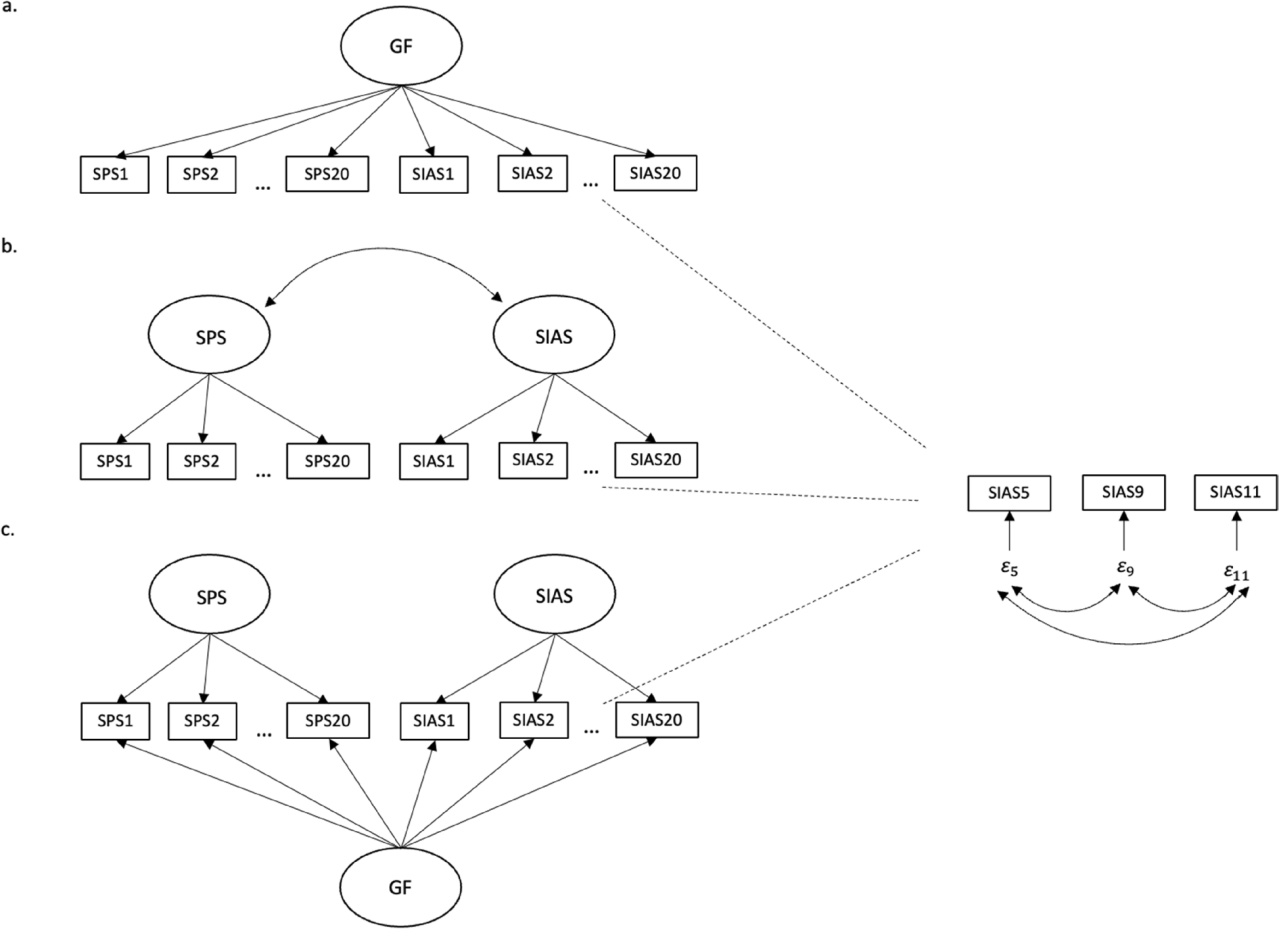
One-factor (**a**), two-factor (**b**), and bifactor model (**c**). SIAS 5, SIAS 9, and SIAS 11 are reversed items; therefore, their errors were additionally correlated in each model (a, b, and c) to account for method variance

Three models were tested: a one-factor, a two-factor, and a bifactor model (see Fig. 1). The one-factor model assumed that one factor for all items (SPS 1–SPS 20, SIAS 1–SIAS 20) adequately represents social anxiety. The two-factor model assumed two factors (SPS and SIAS). All SPS items (SPS 1–SPS 20) were assumed to load on the SPS factor and to measure social performance anxiety and all SIAS items (SIAS 1–SIAS 20) were assumed to load on the SIAS factor and to measure social interaction anxiety. The marker indicators were SPS 1 and SIAS 1. The covariance was not set to 0. The factors were assumed to be oblique, since it was unrealistic for the two factors, which are both supposed to measure social anxiety, not to correlate at all and to be orthogonal. The bifactor model assumed three factors (GF, SPS, and SIAS) and was a combination of the first two models. All SPS items (SPS 1–SPS 20) loaded on the group factor SPS and all SIAS items (SIAS 1–SIAS 20) loaded on the group factor SIAS, while all 40 items simultaneously loaded on GF. The marker indicators were again SPS 1 and SIAS 1. As usual for the bifactor model, covariance was set to 0 for all factors. Thus, they were orthogonal [21].

The Mplus8 base program (Version 1.8.7.; Muthén & Muthén, 1998–2017) was used for the factor analyses. Model estimation was performed using the weighted least square means and variance adjusted (WLSMV) estimator, which is adequate for the categorial items and the lack of normal distribution [22]. The three reversed SIAS items SIAS 5, SIAS 9, and SIAS 11 have been repeatedly found to not belong to the other SIAS items and were assumed to rather measure extraversion [5, 9–12]. Their errors were correlated in all models as it is recommended by Brown [23] for reversed scored items in general. No cross-loadings between the items were allowed in any of the models.

Model fit was assessed through the following indices: Tucker Lewis index (TLI), comparative fit index (CFI), root mean square error of approximation (RMSEA), and standard root mean square residual (SRMR). Following Hu & Bentler [24], TLI and CFI of ≥ 0.90 were considered as acceptable fit and ≥ 0.95 as good fit. RMSEA and SRMR ≤ 0.08 were considered as acceptable fit and ≤ 0.05 as good fit. For RMSEA, Mplus calculates the 90% confidence interval (CI), where the lower CI should not be higher than 0.05 (the closer to 0, the better) and the upper not higher than 0.08 [25].

${\chi }^{2}$ depends on the sample, so it is recommended to use other model indices than solely ${\chi }^{2}$ and to not reject or accept a model based on the significance of ${\chi }^{2}$ only [25, 26]. With this sample size, ${\chi }^{2}$ was expected to be significant (i.e., reject the proposed models) (cf. [27]). Model fit indices for a bifactor model tend to be better in comparison to a first-order factor model, especially if the bifactor model does not reflect the true model [28]. ${\chi }^{2}$ is in this case not a suitable comparison coefficient, therefore, additional coefficients for the evaluation of the bifactor model were used. Even if a bifactor model turns out to have the relatively best fit, it is still not proof of “enough” multidimensionality, nor of useful subscales [29]. The following coefficients were used following Rodriguez et al. [21]: omega ($\omega$), omega hierarchical (${\omega }_{h}$), omega hierarchical subscale (${\omega }_{hs}$), explained common variance (ECV), percent uncontaminated correlations (PUC), factor determinacy (FD), and coefficient H (H) (for further explanation on the individual coefficients, see [21]).

## Results

### Comparisons of sample (a) and (b) and characteristics of the analysis sample

Sample (a) and (b) did not differ significantly in age, sex, education, or relationship status (see Table 1). The average age in sample (a) and (b) combined was 35.1 years (SD = 11.2) with 58% women. The majority of the sample had a tertiary education (70%) and was single (51%). Furthermore, none of the comparisons of the mean responses of the 40 items were significant (see Supplementary file 2, Supplementary Table 1 for all item comparisons). Thus, we combined both samples for the following analyses.

**Table 1:** Socio-demographic information of the samples (a), (b), and the full sample (both samples combined)

	Sample (a)		Sample (b)				Full sample	
	M	SD	M	SD	*U*	*p*-value	M	SD
Age	35.4	11.2	34.8	11.2	11406	.682	35.1	11.2
	Sample (a)		Sample (b)				Full sample	
	n	%	n	%	${\chi }^{2}$	*p*-value	n	%
Sex					2.7	.101		
women	79	53%	93	62%			172	58%
men	70	47%	56	38%			126	42%
Education					1.04	.596		
primary	5	3%	8	71%			13	4%
secondary	41	28%	36	24%			77	26%
tertiary	103	69%	105	5%			208	70%
Relationship status					7.7	.053		
single	77	52%	74	50%			151	51%
married	27	18%	36	24%			63	21%
separated, divorced, widowed	11	7%	2	1%			13	4%
in a relationship (not married)	34	23%	37	25%			71	24%
Total	149		149				298	

Sample (a) = sample from Schulz et al. [16]; Sample (b) = sample from Stolz et al. [17]; *U* = Mann–Whitney U coefficient

### Model comparisons

Table 2 shows the goodness of fit indices and comparisons of all models. As expected, all three models showed a highly significant ${\chi }^{2}$ with *p* < 0.001. The one-factor model (O) did not show any acceptable indices. All items loaded significantly on the factor. The two-factor model (T) showed only an acceptable RMSEA. The remaining indices were not acceptable. The factors SPS and SIAS correlated moderately with *r* = 0.57, which supports the decision to assume an oblique structure.

**Table 2:** Model indices and comparison of the three models

Model	RMSEA [90% CI]	SRMR	CFI	TLI	$\bf {\chi }^{2}$	df
One-factor (O)	0.093 [0.089, 0.097]	0.108	0.719	0.702	2648.812***	737
One-factor^1^ (O1)	0.094 [0.091, 0.098]	0.110	0.711	0.695	2704.934***	740
Two-factor (T)	0.073 [0.069, 0.077]	0.089	0.830	0.820	1891.400***	736
Two-factor^1^ (T1)	0.074 [0.070, 0.078]	0.091	0.825	0.815	1931.936***	739
Bifactor (B)	0.059 [0.054, 0.063]	0.067	0.894	0.882	1416.337***	697
Bifactor^1^ (B1)	0.059 [0.055, 0.063]	0.067	0.893	0.881	1428.522***	700
Bifactor without non-significant elements	0.052 [0.047, 0.056]	0.067	0.917	0.908	1267.480***	704

*RMSEA* Root mean square error of approximation; *SRMR* Standard root mean square residual, *CFI* comparative fit index, *TLI* Tucker Lewis index

^***^ *p* < .001

^1^model without error correlations

The bifactor model (B) showed two acceptable indices, RMSEA and SRMR, but CFI and TLI remained not acceptable. Seven items did not load on the general nor on the specific factors and one error correlation was not significant (*p* > 0.05): For group factor SPS: SPS 3 (*p* = 0.407), and SPS 18 (*p* = 0.207). For GF: SPS 1 (*p* = 0.929), SPS 7 (*p* = 0.359), SPS 10 (*p* = 0.499), SPS 19 (*p* = 0.822), and SIAS 5 (*p* = 0.123). For the error correlation: SIAS 5 × SIAS 11 (*p* = 0.155). A refined bifactor model without the non-significant elements was tested. The modification suggestion, also used by Eidecker et al. [9], to add the correlation between SIAS 12 × SIAS 17, was additionally implemented. All four indices showed acceptable values and all remaining elements were significant. Since most of the other studies initially did not include any error correlations in their models, the same three models were tested again, but without the error correlations (O1, T1, B1). All three models were not better without the error correlations in comparison to with; the same indices remained acceptable respectively not acceptable. The findings [6, 11, 12, 30] that the reversed items should be excluded from the final set of items was only partly shown since only SIAS 5 was not significant. To exclude any potential sample-specific biases, the models were tested separately for sample (a) and sample (b). None of the models with separate samples had more acceptable indices than the models with the combined sample. Additionally, the desired ratio ${\chi }^{2}$/ df < 2 was only found for the bifactor model without the non-significant elements (cf. [8]). No ${\chi }^{2}$ difference tests were reported since they cannot be conducted in a regular way for models based on the WLSMV estimator (cf. [6]).

In conclusion, only the refined bifactor model showed an acceptable, but not a good fit to the data. Moreover, there were numerous non-significant item loadings primarily in the GF. Therefore, we performed EFAs with the same data set.

### Exploratory factor analysis

The requirements for an EFA as well as the estimated factor number were conducted with RStudio and Mplus. For the correlation matrix, which was used for both tests, a spearman correlation was used, since normal distribution was not given, and the spearman correlation is suited for ordinal variables. The rotation bi-geomin for orthogonal structures was used. The Bartlett test of sphericity and the Kaiser–Meyer–Olkin test indicated that the sample was adequate for an EFA (the Bartlett test of sphericity: ${\chi }^{2}$ (780, *N* = 298) = 4651.873, *p* < 0.001; matric sample adequacy (MSA) of the Kaiser–Meyer–Olkin test: 0.88 (range: 0.73 – 0.94), which indicates excellent MSA according to Kaiser [31]).

Since the bifactor model in the CFA was the best one so far, it was assumed that the new model should be a bifactor model as well, but with potentially more than two group factors. The parallel analysis indicated 5 factors. The Kaiser-Guttman rule would have indicated 10 factors (eigenvalue > 1), while the first 3 factors seemed to explain the most variance, with an eigenvalue of 10.8, 4.4 respectively 2.3. All the other factors had an eigenvalue < 2. Moosbrugger & Hartig [32] and Brown [23] indicated that the Kaiser-Guttman rule could lead to over-factorization, which was probably the case here. Items with loadings < 0.30 (i.e., not salient loadings) were excluded [23]. When cross-loadings occurred and the difference between the two loadings was > 0.10, the item with the higher loading was chosen. When the difference was < 0.10, it was considered a cross-loading (cf. [7]).

The following models were tested: bifactor model with two, three, four, and five group factors. In every model, the following same items as in the original bifactor model without the non-significant elements were not significant for GF: SPS 1, SPS 7, SPS 19, and SIAS 5. SPS 10 was also not significant for all models except for the bifactor model with five group factors. All models had between five and eight non-significant items in the GF and between eleven and eighteen non-significant items in the group factors. With an item-driven approach, we were not able to find distinct categories for any of the models. We were not able to find a better-fitting model than the current bifactor model. Therefore, the bifactor-specific indices were calculated for this model.

### Bifactor-specific indices

Table 3 shows the final refined bifactor model including the factor loadings as well as the bifactor model indices. The indices were calculated with the Bifactor Indices Calculator by Dueber [33]. Comparing $\omega$ and ${\omega }_{h}$ of GF showed that 74% (0.7 / 0.94) of the reliable variance in total scores is attributed to GF, while 24% (0.94 – 0.7) of the reliable variance in total scores is attributed to multidimensionality caused by SPS and SIAS. ${\omega }_{s}$ of SPS and SIAS indicated that SPS and SIAS are reliable subscales with 97% (0.91 / 0.94) for SPS and 98% (0.92 / 0.94) for SIAS of the reliable variance being independent of GF [29]. ${\omega }_{hs}$ showed that the common variance explained by the group factors, while controlling for GF, is still relatively high with 52% explained by SPS respectively 47% explained by SIAS, which is further in favor of the reliability for SPS and SIAS as separate reliable subscales to measure the two different aspects of social anxiety.

**Table 3:** Standardized factor loadings of refined bifactor model and bifactor model indices

Item	GF	SPS	SIAS
SPS 1	–	0.44	
SPS 2	0.28	0.20	
SPS 3	0.47	–	
SPS 4	0.58	0.43	
SPS 5	0.38	0.21	
SPS 6	0.61	0.33	
SPS 7	–	0.70	
SPS 8	0.50	0.54	
SPS 9	0.33	0.51	
SPS 10	–	0.70	
SPS 11	0.22	0.57	
SPS 12	0.68	0.16	
SPS 13	0.36	0.55	
SPS 14	0.31	0.41	
SPS 15	0.63	0.30	
SPS 16	0.60	0.39	
SPS 17	0.51	0.53	
SPS 18	0.66	–	
SPS 19	–	0.56	
SPS 20	0.67	0.22	
SIAS 1	0.49		0.17
SIAS 2	0.56		0.19
SIAS 3	0.42		0.44
SIAS 4	0.32		0.39
SIAS 5	–		0.58
SIAS 6	0.42		0.27
SIAS 7	0.43		0.35
SIAS 8	0.29		0.41
SIAS 9	0.22		0.36
SIAS 10	0.40		0.68
SIAS 11	0.19		0.77
SIAS 12	0.58		0.39
SIAS 13	0.34		0.43
SIAS 14	0.40		0.41
SIAS 15	0.52		0.68
SIAS 16	0.49		0.32
SIAS 17	0.58		0.36
SIAS 18	0.42		0.45
SIAS 19	0.62		0.41
SIAS 20	0.51		0.29
omega ($\omega$) / $\omega$ subscale (${\omega }_{s}$)	.94	.91	.92
omega hierarchical (${\omega }_{h}$) / ${\omega }_{h}$ subscale (${\omega }_{hs}$)	.70	.52	.47
ECV	.51	.24	.25
PUC	.42		
FD	.94	.91	.92
H	.92	.85	.86

“– “ for a factor loading means that this loading was not significant and was excluded for the final bifactor model

*GF* General factor, *SPS* SPS group factor, *SIAS* SIAS group factor, *ECV* Explained common variance, *PUC* Percent uncontaminated correlations, *FD* Factor determinacy; *H* Coefficient H

ECV and PUC were below 0.70, indicating that the common variance is not unidimensional respectively a unidimensional model may not be sufficient to represent the data. Additionally, the low PUC indicated probably biased parameters if the model was forced as unidimensional. All three FDs were above 0.9 as well as all three Hs were above 0.7, indicating a good construct reliability and therefore a stable representation of the latent variable by the indicators and a good representation of the two aspects of social anxiety [21].

In conclusion, while GF explained most of the variance (70%) and half of the common variance, the group factors explained equally the remaining common variance and were found to be reliable. There are clear indicators that the bifactor model is tendentially multidimensional.

### Content analysis of final bifactor model

The final bifactor model in Table 3 shows several items which were not significant for the general factor GF and the group factor SPS. Additionally, there were some item loadings in the general as well as in the two group factors that were < 0.30. This section will first, examine the content of the none-significant items and second, the content of the items with item loadings < 0.30.

There were seven items in the group factor SPS and general factor GF, which the item loadings were not significant for. For the group factor SPS, the items SPS 3 (“I can suddenly become aware of my own voice and of others listening to me”) and SPS 18 (“I get tense when I speak in front of other people”) were not significant. The two items refer to aspects of the voice and being listened to respectively public speaking situations in general. For GF, items SPS 1 (“I become anxious if I have to write in front of other people”), SPS 7 (“I worry about shaking or trembling when I'm watched by other people”), SPS 10 (“I would find it difficult to drink something if in a group of people”), SPS 19 (“I worry my head will shake or nod in front of others”), SIAS 5 (“I find it easy to make friends of my own age”) were not significant. SPS 1, SPS 7 and SPS 10 refer to mundane performance tasks, while SPS 19 and SIAS 5 do not match the rest with worrying about losing control and making friends. In summary, there are more items from the SPS questionnaire that do not seem to fit either performance-related social anxiety (i.e., not significant for group factor SPS), or general social anxiety (i.e., not significant for general factor). We were not able to find a common interpretation of the topics covered by said items.

There were twelve item loadings across the two group factors and GF that were below the threshold of < 0.30. Since the loadings are standardized and there were no cross-loadings, the item loadings can be interpreted as correlations between the items and the factors [23]. For the group factor SPS, the item loadings of SPS 2 (“I become self-conscious when using public toilets”), SPS 5 (“I fear I may blush when I am with others”), and SPS 12 (“I am worried people will think my behaviour odd”) were below the threshold. There is no apparent similarity across these three item contents. Therefore, there is a small correlation between social performance anxiety and the fear to blush, public toilet usage, and appearing odd. For the group factor SIAS, the item loadings for SIAS 1 (“I get nervous if I have to speak with someone in authority (teacher, boss, etc.)”), SIAS 2 (“I have difficulty making eye-contact with others”), SIAS 6 (“I tense-up if I meet an acquaintance in the street”), and SIAS 20 (“I am unsure whether to greet someone I know only slightly”) were below the threshold. All the items refer to situations involving people one does not know on a personal level and maintaining eye contact. Therefore, there is a small correlation between social interaction anxiety and situations involving acquaintances and maintaining eye contact. For GF, the item loadings for SPS 2 (“I become self-conscious when using public toilets”), SPS 11 (“It would make me feel self-conscious to eat in front of a stranger at a restaurant”), SIAS 8 (“I feel tense if I am alone with just one other person”), SIAS 9 (“I am at ease meeting people at parties, etc.”), and SIAS 11 (“I find it easy to think of things to talk about”) were below the threshold. While the last four items refer to situations involving strangers, SPS 2 again does not match the rest. Therefore, there is a small correlation between social anxiety in general and situations involving strangers and public toilet usage.

In conclusion, there were, in general, more SPS items than SIAS items that were not significant (six SPS and two SIAS items), which means that social performance anxiety is less well represented in this model than interaction anxiety. Concerning the items below threshold, more SIAS items had only a small correlation than SPS items (four SPS and seven SIAS items). All SIAS items with small correlations revolved around acquaintances, respectively strangers and situations involving talking to others, which implies that situations involving conversations and unknown people represent social anxiety in general and social interaction anxiety less well. SPS 2 was the only item that had a small correlation with both GF and group factor SPS and did not seem to represent social anxiety in general or social performance anxiety well. There was no apparent similarity between all the other SPS items (blushing, eating in front of others, and appearing odd).

## Discussion

SPS and SIAS are one of the most widely used questionnaires to assess social anxiety, but to this day, there is no consensus on their joint factor structure, nor general consensual implications on how to use and evaluate the questionnaires in practice, whether they can be reliably evaluated simultaneously as well as separately. The two aims of this study were to investigate the joint factor structure of the SPS and SIAS and to provide practical implications for the use of the questionnaires based on these results. The one-factor, two-factor, and bifactor models were tested with a CFA and other possible bifactor models were tested with an EFA. Further, bifactor-specific indices as well as item contents were examined.

The best model in comparison with an acceptable fit was the bifactor model without the eight non-significant elements. The corresponding bifactor indices showed that GF, SPS, and SIAS were reliable factors, that SPS and SIAS both explained a high amount of reliable variance independent of GF and that the joint factor structure of SPS and SIAS is tendentially multidimensional. According to these results, the combined use in practice, as well as the report of the two questionnaires separately, is justified. This could not be shown for all items, since seven items in the model were not significant. Thus, the questionnaire SIAS seems to be more fitting to this model since none of the items on SIAS and only one on GF were not significant. One possible explanation is that the non-significant items do not represent the construct performance anxiety accurately. Another explanation comes from Caballo et al. [34], who found that items that referred to cognitive aspect of social anxiety, which is especially true for SPS items, were not always clear to university students and were misunderstood in their study, which could also be the case for this sample. On the other hand, there were more SIAS items than SPS items across GF and the group factors that were below the threshold. Most of the SIAS items revolved around situations involving acquaintances or strangers. There was only a small correlation between these types of situations and interaction respectively general social anxiety. SPS 2 (usage of public toilet) was the only item that was below the threshold for GF as well as for the group factor SPS. This could be an indication that the newly introduced phobia “paruresis” could be considered a separate disorder.

The deviation of the three reversed SIAS items from the other SIAS items could only partly be found in this study since only item SIAS 5 was not significant for GF. Additionally, the item loadings of SIAS 9 and SIAS 11 in GF were below the threshold of 0.30, indicating a small correlation between the two reversed items and general social anxiety.

There are several reasons that could explain the general inconsistent factor solutions across different studies. The first explanation is according to Caballo et al. [35] the non-empirical approach of constructing the questionnaires in the first place, where not enough attention was paid to content validity. The second explanation is that the questionnaires were originally created for English-speaking users and are usually directly translated into other languages without concern for possible cultural differences [35]. Depending on the culture of the sample, the items of the questionnaires may not mean the same for every culture. Carter et al. [30] have demonstrated that the item SPS 5, the fear of blushing, for example, may not be relevant for most of the African American population. The third explanation is according to Caballo et al. [34] the often vague definition of the target group in the SPS and SIAS items. The items ask for example about “people” and “others” which could lead to a broad spectrum of interpretations and in the end not referring to the same situation. The fourth explanation may be the primary focus of the items on cognitive aspect according to Caballo et al. [34], which was mentioned before. Particularly in samples from the community, it may be that some of the items were not understood correctly. The fifth explanation concerns the problem with the number of SIAS items: Some studies use the SIAS questionnaire with 19 items, other the SIAS with 20 items. Along the way, a 20^th^ item was added to the set of Mattick & Clarke [14]. This was also mentioned by Rodebaugh et al. [11], who pointed out that Mattick & Clarke [14] once mentioned 20 items for the SIAS on p. 462, but only reported 19 items at the end. It does not seem to be clear what exactly happened. This makes it further difficult to compare studies.

There are several limitations to this study. Firstly, CFA is a large-sample technique [26]. It may be that the sample size is not big enough to produce robust results for the resulting complex bifactor model. Secondly, no conclusions on the validity of the two questionnaires are possible. Thompson et al. [3] for example do not support the use of SPS and SIAS as separate questionnaires to assess additional to general social anxiety performance respectively interaction anxiety due to their insufficient discriminant criterion validity. This is to our knowledge the only study that tested the validity based on behavioral tests (i.e., inducing performance respectively interaction anxiety). The validity would need to be tested in future clinical studies, especially the construct validity since it assumed that SPS measures performance and SIAS interaction anxiety, but there are almost no studies that tested these assumptions with actual behavioral tests as Thompson et al. [3] did. In general, all the studies that did not recommend the separate use of SPS and SIAS were based on non-clinical samples. It may also be that the clinical population has a more clear and distinct picture of social anxiety. Thirdly, the sample is self-selective as participants are self-referred to the intervention, were prepared to download the study information from a website and send the ICF back. This suggests that participants may have had a high level of motivation to seek treatment. Also, while age, relationship status, and the balanced sex were comparable to the typical clinical SAD population (see [13]), the education level was higher. We can additionally speculate that the sample showed a higher psychosocial functioning than other clinical samples since the participants were able to work through an online therapy program with minimal guidance. These factors might have influenced the external validity of this study. Fourthly, the goodness of fit indices (i.e., RMSEA, SRMR, CFI, TRI) of the final refined bifactor model are only acceptable. This may reflect the overall inconsistent current literature on the joint factor structure as well as on the separated factor structure of SPS and SIAS. Mattick & Clarke [14] found, when developing the questionnaires, a three-factor structure for SPS and a one-factor structure for SIAS, while the literature overview in the introduction of e.g., Gomez & Watson [5] and Eidecker et al. [9] shows inconsistent results for either questionnaire over different studies.

## Conclusion

The findings of this study show that SPS and SIAS are reliable questionnaires to assess specific aspects of social anxiety beyond the assessment of social anxiety in general. It still needs to be examined whether SPS measures social performance anxiety and SIAS social interaction anxiety in a clinical sample as claimed. Future studies with a clinical sample recruited from the community could try to replicate the results and test whether the bifactor model is the relatively best model and whether the same items turn out to not be significant. Social performance anxiety, measured with the SPS, does not seem to be as well represented by the model as social interaction anxiety, measured by the SIAS. It would be interesting to see, whether it is a cultural influence or due to a selection bias from the self-referred sample.

In conclusion, this study shows that the joint factor structure of SPS and SIAS is potentially multidimensional and that they are indeed reliable questionnaires to assess specific aspects of social anxiety beyond the assessment of social anxiety in general.

### Supplementary Information

**Additional file 1: Supplementary File 1.** Item wording.

**Additional file 2: Supplementary Table 1.** Comparison of item responses between sample (a) & sample (b).

## Data Availability

The datasets used and analyzed during the current study are available from the corresponding author on reasonable request.

## Abbreviations

B: Bifactor model
B1: Bifactor model without the error correlations
CI: Confidence interval
CFA: Confirmatory factor analysis
CFI: Comparative fit index
DSM-5: 5^Th^ edition of the Diagnostic and Statistical Manual of Mental Disorders
ECV: Explained common variance
EFA: Exploratory factor analysis
FD: Factor determinacy
GF: General factor
H: Coefficient H
ICBT: Internet-based cognitive behavioral therapy
ICF: Informed consent form
O: One-factor model
O1: One-factor model without the error correlations
PUC: Percent uncontaminated correlations
RCT: Randomized controlled trial
RMSEA: Root mean square error of approximation
SAD: Social anxiety disorder
SCID: Structured Clinical Interview for DSM-IV – Axis I disorder
SIAS: Social interaction anxiety scale
SPS: Social phobia scale
SRMR: Standard root mean square residual
T: Two-factor model
T1: Two-factor model without the error correlations
TLI: Tucker Lewis index
*U*: Mann-Whitney U coefficient
WL: Waitlist control group
