# Supplementary material for: Factor structure of the Social Phobia Scale (SPS) and the Social Interaction Anxiety Scale (SIAS) in a clinical sample recruited from the community
Source: BMC Psychiatry. 2023 Sep 4;23:646. doi: 10.1186/s12888-023-05142-8 (PMC10478387; doi:10.1186/s12888-023-05142-8)
Supplement: Supplementary file 1 — Additional file 1: Supplementary File 1. Item wording. [file 12888_2023_5142_MOESM1_ESM.pdf]

Supplementary File 1

Item wording

SPS item wording (14)

1. I become anxious if I have to write in front of other people
2. I become self-conscious when using public toilets
3. I can suddenly become aware of my own voice and of others listening to me
4. I get nervous that people are staring at me as I walk down the street
5. I fear I may blush when I am with others
6. I feel self-conscious if I have to enter a room where others are already seated
7. I worry about shaking or trembling when I'm watched by other people
8. I would get tense if I had to sit facing other people on a bus or a train
9. I get panicky that others might see me to be faint, sick or ill
10. I would find it difficult to drink something if in a group of people
11. It would make me feel self-conscious to eat in front of a stranger at a restaurant
12. I am worried people will think my behaviour odd
13. I would get tense if I had to carry a tray across a crowded cafeteria
14. I worry I'll lose control of myself in front of other people
15. I worry I might do something to attract the attention of others
16. When in an elevator I am tense if people look at me
17. I can feel conspicuous standing in a queue
18. I get tense when I speak in front of other people
19. I worry my head will shake or nod in front of others
20. I feel awkward and tense if I know people are watching me

SIAS item wording (14)

1. I get nervous if I have to speak with someone in authority (teacher, boss, etc.)
2. I have difficulty making eye-contact with others
3. I become tense if I have to talk about myself or my feelings
4. I find it difficulty mixing comfortably with the people I work with
5. I find it easy to make friends of my own age [a]
6. I tense-up if I meet an acquaintance in the street
7. When mixing socially I am uncomfortable
8. I feel tense if I am alone with just one other person

9. I am at ease meeting people at parties, etc.
10. I have difficulty talking with other people
11. I find it easy to think of things to talk about
12. I worry about expressing myself in case I appear awkward
13. I find it difficult to disagree with another's point of view
14. I have difficulty talking to attractive persons of the opposite sex
15. I find myself worrying that I won't know what to say in social situations
16. I am nervous mixing with people I don't know well
17. I feel I'll say something embarrassing when talking
18. When mixing in a group I find myself worrying I will be ignored
19. I am tense mixing in a group
20. I am unsure whether to greet someone I know only slightly

Note. [a] item content was taken from Safren et al. (7), since the paper from Mattick & Clarke (14) does not contain this item.
