# Supplementary material for: Factor structure of the Social Phobia Scale (SPS) and the Social Interaction Anxiety Scale (SIAS) in a clinical sample recruited from the community
Source: BMC Psychiatry. 2023 Sep 4;23:646. doi: 10.1186/s12888-023-05142-8 (PMC10478387; doi:10.1186/s12888-023-05142-8)
Supplement: Supplementary file 2 — Additional file 2: Supplementary Table 1. Comparison of item responses between sample (a) & sample (b). [file 12888_2023_5142_MOESM2_ESM.pdf]

Supplementary Table 1

Comparison of item responses between sample (a) & sample (b)

SPS Item Nr.	Sample (a)		Sample (b)		*U*	*p(U)*
	Mean	SD	Mean	SD		
**1**	1.47	1.11	1.4	1.18	11633	.453
**2**	0.9	1.11	1.01	1.11	10325	.266
**3**	2.01	1.36	2.03	1.13	11004	.894
**4**	1.78	1.31	1.73	1.29	11308	.774
**5**	1.81	1.54	1.79	1.44	11172	.921
**6**	2.84	1.1	2.7	1.07	11949	.235
**7**	1.93	1.45	1.8	1.56	11771	.355
**8**	1.77	1.37	1.74	1.33	11192	.900
**9**	1.01	1.35	1.24	1.48	10194	.189
**10**	0.97	1.15	0.97	1.22	11188	.900
**11**	1.68	1.34	1.68	1.41	11134	.964
**12**	2.97	1.07	3.07	1.02	10543	.427
**13**	2.08	1.29	2.17	1.36	10675	.558
**14**	2	1.31	2.13	1.36	10487	.398
**15**	2.75	1.08	2.66	1.18	11462	.614
**16**	2.21	1.31	2.08	1.27	11781	.348
**17**	1.58	1.33	1.58	1.39	11219	.870
**18**	3.45	0.9	3.43	0.83	11511	.523
**19**	0.59	1.14	0.74	1.25	10392	.245
**20**	3.2	0.99	3.24	1.09	10500	.373
SIAS Item Nr.	Sample (a)		Sample (b)		*U*	*p(w)*
	Mean	SD	Mean	SD		
**1**	2.91	1.12	3.05	1.03	10381	.307
**2**	2.07	1.33	2.15	1.28	10698	.579
**3**	2.45	1.26	2.64	1.28	10072	.154
**4**	1.68	1.26	1.6	1.11	11341	.738
**5**	3.15	1.03	3.14	1.06	11054	.946
**6**	2.26	1.25	2.26	1.35	11104	.997
**7**	2.01	1.11	1.97	1.02	11177	.914
**8**	1.83	1.29	1.82	1.29	11140	.956
**9**	3.27	0.89	3.12	1.03	11776	.326
**10**	2.35	1.22	2.21	1.23	11825	.317
**11**	3.17	1.05	3.24	0.96	10805	.665
**12**	2.76	1.14	2.85	1.08	10638	.517
**13**	2.13	1.29	2.11	1.35	11171	.923
**14**	2.64	1.3	2.58	1.32	11380	.698
**15**	2.89	1.26	3.03	1.17	10510	.399

**16**	2.84	1.09	2.91	0.98	10888	.765
**17**	2.41	1.33	2.46	1.28	10893	.775
**18**	2.42	1.33	2.4	1.34	11187	.906
**19**	3.25	0.9	3.26	0.89	11011	.896
**20**	2.32	1.48	2.13	1.45	11954	.240

Note. Sample (a) = sample from Schulz et al. (16); Sample (b) = sample from Stolz et al. (17)
